# Health Service Early-Stage Digital Adaptation of Traditional Chinese Medicine Internet Hospitals: Qualitative Exploratory Study

**DOI:** 10.2196/77686

**Published:** 2025-11-05

**Authors:** Yao Wang, Menghuan Song, Zhenmiao Pang, Buping Liu, Dongning Yao, Meng Li, Zhirong Xie, Ying Bian, Hao Hu, Yunfeng Lai

**Affiliations:** 1 State Key Laboratory of Mechanism and Quality Research in Chinese Medicine, Institute of Chinese Medical Sciences, University of Macau, Macao, China; 2 Centre for Pharmaceutical Regulatory Sciences, University of Macau, Macao, China; 3 School of Public Health and Management, Guangzhou University of Chinese Medicine, No. 232, Huandong Road, University City, Panyu District, Guangzhou, 511400, China, 86 020-39358970; 4 School of Pharmacy, Nanjing Medical University, Nanjing, China; 5 Key Laboratory of Environmental Medicine Engineering of Ministry of Education, Department of Medical Insurance, School of Public Health, Southeast University, Nanjing, China; 6 Department of Science and Technology, Guangzhou University of Chinese Medicine, Guangzhou, China; 7 Department of Public Health and Medicinal Administration, Faculty of Health Sciences, University of Macau, Macao, China

**Keywords:** traditional Chinese medicine, internet hospital, health service, target patient, value offering

## Abstract

**Background:**

Traditional Chinese medicine (TCM) hospitals in China are experimenting to develop internet hospitals to provide health services. To date, little is known about the characteristics of health services delivered by TCM internet hospitals.

**Objective:**

This study aimed to investigate the health service early-stage digital adaptation of TCM internet hospitals from the aspects of target patients, value offering, and service provision.

**Methods:**

Qualitative research combined qualitative interview and documentary research in this study. Interviews were completed with clinicians from sample TCM internet hospitals to investigate the target patients and value offerings. Documentary research was conducted to investigate the service provision. Thematic analysis was used to interpret all the materials collected.

**Results:**

A total of 7 TCM internet hospitals and 14 participants were included. The target patients of TCM internet hospitals were patients with subsequent visits and patients who sought consultations on health management. TCM internet hospitals were improving patients’ adherence to subsequent medical care and TCM promotion. These hospitals provided functional service (including telemedicine, telepharmacy, telenursing, web-based health consultations, and convenient service), and TCM specialty service (including “Tianzhi” [crude herb moxibustion], “Zhiweibing” [preventive treatment of disease], and poststroke rehabilitation).

**Conclusions:**

TCM internet hospitals are in an early-stage digital adaptation, offering primarily basic online-offline services. While not yet fully innovative, they represent a transitional model with the potential to reshape TCM delivery. Our findings contribute high-level insights into this emerging integration and inform future development toward more structured, patient-centered digital TCM services.

## Introduction

### Background

Digital technology has exceptional advantages for disease prevention and treatment. The advantages are improving the feasibility of health information collection and patient-centered care, enhancing the efficiency of monitoring, diagnoses, and prognoses of diseases, upgrading medical services, and reducing service costs [1]. In the context of digitalization, the Chinese government is actively promoting the integration of the *internet* and *health care* [2]. Internet hospitals rely on brick-and-mortar hospitals as a base and benefit from internet technology and health resources of their physical hospitals and provide patients with closed-loop medical services [3]. Internet hospitals, guided by government policy, are rapidly expanding to enhance access to quality health care for patients with common or chronic conditions and those in remote areas, thereby improving resource accessibility and saving time for patients and their families [4].

In China, traditional Chinese medicine (TCM) hospitals have evolved into institutions offering comprehensive and diversified TCM services and products, including Chinese patent medicines, herbal pieces, formula granules, and raw herbs [5]. As one of the oldest therapeutic systems in the world, TCM is gaining increasing attention from global society [6]. In the background of digitalization, TCM hospitals also dived into the development of web-based service platforms. Until August 2021, there were more than 1600 internet hospitals across China, of which about 200 were TCM internet hospitals located in Guangdong, Shandong, and Jiangsu provinces [7,8]. TCM internet hospitals have emerged as a vital component of modern health care, especially in China, where digital health initiatives are rapidly transforming the medical landscape. These hospitals integrate TCM with cutting-edge digital technologies, providing web-based registration, health consultation, prescriptions, and payment services based on TCM theories and practices. Although the amount of health resources allocated to TCM hospitals is increasing [9], the efficiency of their service is unsatisfactory [10]. TCM Internet hospitals, which have the integration of TCM and digitalization, are amenable to offering efficient service [11]. Unlike conventional biomedicine, TCM emphasizes preventive care and individualized diagnosis through pattern differentiation. These features present unique opportunities for digital health innovation, such as personalized service design and algorithm-enabled diagnostic support, which remain underexplored in current TCM internet hospital models.

As an emerging model, “internet+TCM health care” has demonstrated significant advantages in enhancing public accessibility and promoting the rational distribution of TCM medical resources. It has increasingly become a crucial supplementary component to offline TCM clinical services and an integral part of China’s health care system [12]. Moreover, it can also develop internet+TCM home care services, which is good for improving the quality of life of older adult patients with chronic diseases [13]. For the development of TCM internet hospitals, there are many practical experiences that can be promoted, including (1) “internet hospitals+master of TCM” will be the preferred path for TCM hospitals to innovate and create distinctive TCM services; (2) TCM internet hospitals can be used to expand the scope of TCM health management services for preventing diseases; and (3) the development of TCM internet hospitals is conducive to the sustainable development of TCM services [14]. TCM internet hospitals have partially met patients’ demands for web-based health care services. However, their overall implementation remains limited, lacking distinctive TCM characteristics and demonstrating a low degree of integration with offline medical services [15,16]. It should also be noted that due to the lack of appropriate “internet+ health care” items, the use rate of internet medical care in grassroots TCM medical institutions is relatively low [17]. In addition, TCM mobile medical apps represent a specific developmental form of the “internet+ TCM” model. While it highlights the distinctive role of TCM in disease treatment, prevention, and health maintenance, there remains significant room for improvement in areas such as information service models, functional applications, and service content [18].

Investigation of health services of TCM internet hospitals is important for gaining knowledge of the characteristics of the web-based service and stimulating improvement in service quality and efficiency [19]. The recent studies mainly focus on the evaluation index system of information service quality of TCM internet hospitals [20], the resource guarantee, service pricing, service accessorily, and data information security of TCM internet hospitals [21]. The research questions that need to be checked are: What are the characteristics of the health services delivered by the TCM internet hospitals? What are the health services early-stage digital adaptation of TCM internet hospital?

### Objectives

While existing literature and policy documents outline the expansion of digital TCM services, few studies have critically examined how these developments are operationalized within TCM internet hospitals, nor have they assessed whether such services reflect true health service innovation beyond digitized access. This study addresses this gap by investigating the health service early-stage digital adaptation of TCM internet hospitals from the aspects of target patients, value offering, and service provision. The findings of this study are anticipated to offer insights into the integration of TCM and digitalization. It is expected to provide empirical references for personalized digital health services and patient-centered care in TCM internet hospitals, thereby promoting the inheritance and development of TCM.

The conceptual basis of this study. As leading scholars in the field of business model research, Johnson et al [22] argued that a business model consists of four interrelated elements that collectively create and deliver value: (1) value proposition (how TCM internet hospitals enhance patient services); (2) key resources; (3) key processes refer to the operational and managerial aspects and assets necessary for delivering the value proposition; and (4) profit formula (delineate how TCM internet hospitals create and deliver value to both themselves and their patients). Target customer, value offering, and service provision are 3 elements of a value proposition in business model theory [22]. Value offering refers to the important problems or needs of the target customer that are solved or fulfilled, and service provision refers to what specific services are provided. In this study, the value proposition pertains to how TCM internet hospitals enhance patient services. The health service early-stage digital adaptation of TCM internet hospitals can be categorized based on the following 3 aspects: the target patients of TCM internet hospitals, the value offering of TCM internet hospitals, and the service provision of TCM internet hospitals [4].

## Methods

### Research Design

This study conducted a qualitative case study design combining qualitative interviews with participants and documentary research, as TCM internet hospitals are still an emerging sector of web-based health service [19,23]. In this study, we first sampled TCM internet hospitals and described their characteristics. Then, to prepare the materials, interviews were conducted with clinicians experienced in web-based TCM service, and any information posted by sample hospitals on official service platforms (such as WeChat [Tencent], app, and website) was investigated. Data were extracted by reviewing ready materials, including information related to health services (target patients, value offering, and service provision). Finally, data were analyzed, categorized, and assigned themes. Key findings were distilled.

### Research Site: Samples of TCM Internet Hospitals

Internet hospitals in China are all built on the basis of brick-and-mortar hospitals. The sample collection was located in the city of Guangzhou due to its advances in the development of TCM internet hospitals. Data sources were the group of TCM internet hospitals registered at the Guangzhou Municipal Health Commission. Until November 1, 2023, there are a total of 12 TCM hospitals in Guangzhou, all of which claim to provide internet hospital services. We screened the registered hospitals against a set of predetermined selection criteria. TCM internet hospitals were included if: (1) they were established based on Internet Hospital Management Measures (trial) which is a compulsive policy to standardize web-based diagnosis and treatment behaviors, improve medical service efficiency, ensure medical service quality and safety, were general TCM hospitals or hospitals of integrated TCM and western medicine which principally practice TCM; and (2) they were capable of providing medical services via the WeChat platform or app. Finally, 7 TCM internet hospitals met the inclusion criteria. The official webpages of sample hospitals were investigated to extract characteristics, including names of the TCM internet hospitals and names, types (for example, TCM general hospitals and hospitals of integrated TCM and western medicine), grades, business nature (for profit or nonprofit), and the number of beds of the associated brick-and-mortar hospitals. These characteristics were tabulated.

This study was conducted in Guangzhou, one of the most advanced regions in the development of internet-based TCM services in China. A maximum variation sampling strategy was applied to include 7 tertiary-level hospitals with different ownership structures, institutional types, and service models. This approach sought to capture heterogeneity within a single regulatory environment. Although geographically restricted, this design enhances the transferability of findings by providing insights from a leading context of digital TCM implementation.

### Data Collection

#### Qualitative Interviews

Purposive sampling was conducted to select participants who represented the health services of the sample TCM internet hospitals in this study. Prior to sampling, we consulted the medical administrators of each selected TCM internet hospital to determine the clinical departments that deliver the highest volume of medical services within the internet hospital setting. Subsequently, we selected participants from the corresponding clinical departments. We ensured the recruitment of at least 1 participant from each sampled hospital, respectively. Participants with at least 2 years of web-based service experience were approached. The sample size was determined based on the principle of data saturation. We monitored thematic emergence during the interview process using an iterative coding approach. After each round of interviews, 2 researchers independently conducted preliminary coding to identify new themes. Saturation was considered reached when no new codes or themes emerged from at least 2 consecutive interviews. In this study, after the twelfth interview, no additional themes were generated, and the final 2 interviews only confirmed previously identified themes, indicating that thematic saturation had been achieved. The interviews were conducted between November 2023 and December 2023 and adopted a semistructured format by following our tailor-made interview outline.

There is no prior association between the researchers and participants. Before conducting the interviews, we obtained informed consent from the participants. The informed consent forms and interview schedule can be found in MultimediaAppendices 1  2. All interviews involved questions centered on target patients and the value offerings of TCM internet hospitals, and interviewees’ perceptions of the service provisions of TCM internet hospitals and traditional brick-and-mortar hospitals. We recorded the audio of each interview with the participants’ consent. In the process of converting the audio recording into transcripts, two researchers first transcribed the recording independently, then compared the results and marked the unclear and different sections. A third researcher reviewed these marked sections against the recording. Finally, if any ambiguities remained, we verified the unclear parts with the participant. In total, 8 interviews were conducted face-to-face, and 6 interviews were conducted via telephone. The average interview time was about 40 (SD 5.0709) minutes.

#### Documentary Data Collection

When we examined each sample TCM internet hospital one by one, we found that they all used WeChat as the service platform to provide health services. Data were collected from the official WeChat platforms and linked mini programs of the selected TCM internet hospitals. Specifically, WeChat mini programs refer to the *subapplications* within the WeChat ecosystem that enable the provision of advanced features to patients, such as service content, target patients, prices, and other services. To ensure data validity, all WeChat-based information was extracted directly from the official mini programs linked to each hospital’s verified WeChat account and double-checked by 2 researchers independently. While minor differences in interface design and service presentation existed across platforms, we focused our analysis on standardized data elements (eg, service items, target population, and pricing) that were consistently available across hospitals. The last search was conducted on November 15, 2023.

#### Data Privacy and Security

To protect participant confidentiality, personally identifiable information was neither collected nor retained. All interview audio recordings and transcripts were anonymized by assigning unique study codes. Documentary data obtained from WeChat platforms were also screened to remove any patient-identifiable details. All data were stored on encrypted, password-protected institutional servers, with access strictly limited to authorized members of the research team. In compliance with institutional policies, research data will be retained for 5 years after study completion and then permanently deleted.

### Data Analysis

All the interview records were transcribed and stored in a Microsoft Excel–based tool. The thematic analysis approach was applied to the data analysis in steps, including data familiarization, coding, identification of candidate themes, review and revision of themes, definition and naming of themes, and analysis and interpretation of patterns across the data. Two researchers first completed theme identification separately to gain a preliminary understanding of the target patients, value offerings, and service provisions of TCM internet hospitals. Theme saturation was reached through an interactive coding process. Then, their results were compared to examine similarities and differences. If any inconsistency was present, a triangular test was used by 2 other researchers to analyze the differences and decide the definition and naming of the themes.

Besides, thematic analyses were also applied to documentary data extracted from WeChat interfaces. An inductive approach was applied to analyze the health services of TCM internet hospitals. Two independent reviewers scrutinized the data to identify common topics of the target population, service items, service process, and service guidelines. Then, the topics were coded. Patterns of the codes were identified, and themes were generated. Another researcher reviewed the codes and themes and corrected them if feasible. Finally, documentary data were triangulated with interviews based on the review and discussions by all the researchers together to reach an agreement on the final vision of themes.

### Ethical Considerations

This study received ethical approval from the University of Macau Ethics Committee (approval: SSHRE21-APP027-ICMS). All interview participants provided written informed consent prior to participation. No financial or material compensation was provided to the interview participants, as their participation was entirely voluntary and based on their professional interest in the research topic. Data were anonymized, securely stored, and accessible only to authorized researchers. Documentary data were obtained exclusively from publicly available institutional platforms and did not involve identifiable personal information.

## Results

### Characteristics of TCM Internet Hospitals and Participants

A total of 7 TCM internet hospitals were included in this study (Table 1), including 4 (57%) comprehensive TCM hospitals, 2 (29%) integrated TCM and western medicine hospitals, and 1 (14%) specialized TCM hospital. All the internet hospitals were Grade A tertiary hospitals, which is the highest in the Chinese hospital grading system and is scored depending on the establishment of specialty departments, equipment of health professionals and facilities, patient flow, accessibility of beds, and scientific research achievements [24]. From the 7 TCM internet hospitals, 6 (86%) were nonprofit, while 1 (14%) was for profit. The hospital with the largest number of beds was the First Affiliated Hospital of Guangzhou University of Chinese Medicine (2200 beds), which was followed by Guangdong Provincial Hospital of Chinese Medicine (2089 beds) and Guangdong Second TCM Hospital (1490 beds).

**Table 1.** Characteristics of sample traditional Chinese medicine (TCM) internet hospitals (N=7).

	Name of physical hospitals	Name of internet hospitals	Types of hospitals	Grades	Business nature	Beds
1	The First Affiliated Hospital of Guangzhou University of Chinese Medicine	Internet Hospital–The First Affiliated Hospital of Guangzhou University of Chinese Medicine	Comprehensive TCM hospital	Grade A tertiary hospital	Nonprofit	2200
2	Guangdong Second TCM Hospital	Internet Hospital–Guangdong Second TCM Hospital	Comprehensive TCM hospital	Grade A tertiary hospital	Nonprofit	1490
3	Guangdong Provincial Hospital of Chinese Medicine^a^	Internet Hospital–Guangdong Provincial Hospital of Chinese Medicine	Comprehensive TCM hospital	Grade A tertiary hospital	Nonprofit	2089
4	The Third Affiliated Hospital of Guangzhou University of Chinese Medicine	Internet Hospital–The Third Affiliated Hospital of Guangzhou University of Chinese Medicine	Specialized TCM hospital	Grade A tertiary hospital	Nonprofit	800
5	Integrated Hospital of TCM, Southern Medical University	Internet Hospital–Integrated Hospital of TCM, Southern Medical University	Integrated TCM and western medicine hospital	Grade A tertiary hospital	Nonprofit	800
6	Clifford Hospital	Internet Hospital–Clifford Hospital	Integrated TCM and western medicine hospital	Grade A tertiary hospital	For profit	1352
7	The Affiliated TCM Hospital of Guangzhou Medical University	Internet Hospital–The Affiliated TCM Hospital of Guangzhou Medical University	Comprehensive TCM hospital	Grade A tertiary hospital	Nonprofit	722

a Guangdong Provincial Hospital of Chinese Medicine has 3 sites. They are the main site of Guangdong Provincial Hospital of Chinese Medicine, University Town Hospital of Guangdong Provincial Hospital of Chinese Medicine, and Ersha Island Hospital of Guangdong Provincial Hospital of Chinese Medicine. The Internet Hospital–Guangdong Provincial Hospital of Chinese Medicine is the online platform for all the 3 physical sites.

A total of 14 participants took part in the interviews, and their characteristics are presented in Table 2. The specialties of interviewees were various, including cardiologists, pulmonologists, orthopedists, gastroenterologists, gynecologists, obstetricians, physiatrists, and dermatologists. From the 14 participants, 8 (57%) were females, 2 (14%) had master’s degrees, and 12 (86%) had doctoral degrees. The average clinical experience was 9.5 (SD 6.5109) years, and the average experience in providing web-based services was 4.3 (SD 1.1605) years.

**Table 2.** Characteristics of doctors (n=14).

Code	Interviewees^a^	Male or female	Education	Working in hospitals (y)	Working in internet hospitals (y)
1	Cardiologist A	Male	Master’s	8	4
2	Cardiologist B	Female	Doctor	7	4
3	Pulmonologist A	Male	Doctor	5	5
4	Pulmonologist B	Female	Master’s	7	3
5	Orthopedist A	Male	Doctor	4	4
6	Orthopedist B	Male	Doctor	12	5
7	Gastroenterologist A	Male	Doctor	20	6
8	Gastroenterologist B	Male	Doctor	4	4
9	Gynecologist A	Female	Doctor	22	6
10	Gynecologist B	Female	Doctor	7	4
11	Obstetrician A	Female	Doctor	22	6
12	Obstetrician B	Female	Doctor	4	4
13	Physiatrist	Female	Doctor	6	3
14	Dermatologist	Female	Doctor	5	2

a Labels A and B are used to distinguish between different interview participants, representing different interviewees in the same clinical department.

### Target Patients and Value Offerings of TCM Internet Hospitals

#### Target Patients of TCM Internet Hospitals

Return patients with chronic or stable conditions emerged as the primary users of TCM internet hospitals. These patients required follow-up care after initial visits to the physical hospitals. As 1 participant noted:

If they are not return patients, even if they have made an appointment at the internet hospital, we generally cannot provide telemedicine to them because we do not know their conditions. We would suggest them to have an outpatient visit in the first place.

[Interviewer 3]

This reflects the regulatory restriction that web-based consultations are limited to patients with existing medical records, ensuring safety and continuity of care.

A key functional need for these return patients was prescription refills and access to hospital preparations without repeat physical visits, as highlighted below:

Many patients come here (the TCM internet hospital) to refill their prescriptions and buy hospital preparations. The prescriptions can be mailed to the patients. So the patients do not have to come back to the physical hospitals for pick-up.

[Interviewer 4]

This practice reduces the travel burden for patients and improves adherence to long-term treatment plans.

Beyond routine follow-up for common conditions, TCM internet hospitals were also used for postoperative recovery management for patients with tumors:

Aside from patients having colds, cough, hypertension and diabetes, patients with tumours are also accessing TCM internet hospitals. For example, patients who have undergone gastric cancer surgery usually require a long-term follow-up after the discharge. We (doctors of TCM internet hospitals) can adjust the prescriptions depending on patients’ feedback of eat and sleep conditions.

[Interviewer 8]

This illustrates an emerging but important role of TCM internet hospitals in supporting long-term recovery and personalized rehabilitation care.

Consultations for disease prevention and health management represented another group of target users, reflecting growing public awareness of proactive health maintenance. As described below:

There is a large number of people seeking for advices on disease prevention and helaht proection. Nowadays, people generally have good health awareness. Some people accessed the web-based conslutaiton service for themselves; some for family members. Issues of consultaiotns are usually about keeping diets; and managing osteoporosis, cerebral infarction and stroke in the elderly. We are receiving more and more consulotations on strategies of TCM helath protection.

[Interviewer 5]

In addition, younger users, particularly female students, sought advice on gynecological health and sensitive issues, demonstrating the privacy advantage of web-based consultations:

Many young patients, particularly female students, access the web-based service for gynaecological issues, such as abnormal menstruation and excessive leukorrhea. Some people seek for advice on management of unplanned pregnancies. They must be students. They do not know how to deal with accidental pregnancy and are worried about the impact of potential measures on future fertility, so they consult via internet hospital platforms.

[Interviewee 9]

In summary, TCM internet hospitals primarily serve return patients with chronic or stable conditions, individuals seeking preventive care, and younger users requiring sensitive consultations. This reflects a dual role in both clinical follow-up and health management.

#### Value Offerings of TCM Internet Hospitals

One of the most prominent value offerings was improving patient compliance with follow-up care. Remote follow-ups can maintain treatment continuity and reduce the risk of incomplete care. By reducing geographic and time barriers, TCM internet hospitals facilitated more consistent follow-up:

Many patients with bronchitis and coughs are recommended to come back to hospitals for subsequent reviews after a course of treatment which usually takes about 5 or 6 days. But, some patients do not come back to physical hospitals for follow-ups once their symptoms have eased off, because they want to save efforts from accessing offline service…. Nowadays, web-based service can support the consultations on patients’ phlegm, voice, frequency of coughs, and if they have sore throat. In this way, doctors can detect the recovery status of these patients.

[Interviewer 3]

Similarly, privacy-sensitive conditions were more likely to be managed through web-based services, which further promoted adherence:

Some patients with gynaecological diseases, mainly those who are not in serious conditions, are prone to choose internet hospitals for follow-up service. Web-based service is timesaving. Many patients also consider Internet hospitals as a platform good for privacy protection.

[Interviewee 10]

Another key value offering was TCM promotion and patient education. Web-based services enhanced accessibility to renowned TCM practitioners, thereby raising public awareness:

Compared to western medicine practitioners, the number of registered TCM practitioners is smaller, let alone the renowned TCM practitioners. TCM internet hospitals are equipped with famous TCM practitioners, which advanced the access and quality of TCM service.

[Interviewer 2]

Moreover, web-based consultations shifted patients from passive treatment-seeking to proactive health preservation:

In the past years, people are prone to have self-medications when catching colds and coughs with mild symptoms, rather than visiting doctors in clinics. But, health literacy of general population have optimized. With the increasing health awareness, more and more people pay great attention to health preservation. It is very convenient and low costing to find a TCM practitioner to advice on health preservation through WeChat.

[Interviewee 9]

Collectively, TCM internet hospitals deliver value by improving compliance with long-term care, enhancing privacy for sensitive conditions, and serving as a key channel for TCM health promotion and preventive care.

### Health Services of TCM Internet Hospitals

As indicated in Table 3, web-based service items provided by the enrolled hospitals can be divided into 2 categories and 6 subcategories according to the service content. The first category was functional service, which comprised telemedicine, telepharmacy, telenursing, web-based health consultations, and convenient service. All 7 internet hospitals provided functional service. But these hospitals performed differently in the 5 subcategories of functional service. Specifically, the number of sample hospitals offering telemedicine (7/7, 100%), telepharmacy (4/7, 57%), web-based health consultations (3/7, 43%), convenient service (3/7, 43%), and telenursing (2/7, 29%) was in a decreasing trend. The second category of service items was TCM specialty service, which was based on TCM theory and practice. TCM specialty service was delivered by integrating web-based and offline approaches in 3 (43%) hospitals.

**Table 3.** Service items provided by traditional Chinese medicine (TCM) internet hospitals.

	Functional service					TCM specialty service
	Telemedicine	Telepharmacy	Telenursing	Web-based health consultations	Convenient service	
Internet Hospital–The First Affiliated Hospital of Guangzhou University of Chinese Medicine	✓				✓	✓
Internet Hospital–Guangdong Second TCM Hospital	✓					
Internet Hospital–Guangdong Provincial Hospital of Chinese Medicine	✓	✓				
Internet Hospital–The Third Affiliated Hospital of Guangzhou University of Chinese Medicine	✓	✓		✓		✓
Internet Hospital–Integrated Hospital of TCM, Southern Medical University	✓	✓	✓	✓	✓	✓
Internet Hospital–Clifford Hospital	✓	✓	✓	✓	✓	
Internet Hospital–The Affiliated TCM Hospital of Guangzhou Medical University	✓					

#### Functional Service

Telemedicine was the internet hospitals’ core functional service, with the characteristics shown in Table 4. The target patients of telemedicine were mainly patients who had subsequent visits after primary offline medical service. Names of telemedicine in the sample hospitals were various and can be classified into 2 streams. One stream is determining the name by combining terms of *internet* and *medical service* such as *web-based clinic* (2/7, 29%) and *web-based diagnosis and treatment* (1/7, 14%). The other stream (2/7, 29%) used the names consistent with traditional offline medical services provided in the physical hospitals. The examples of this stream were *expert clinic* (1/7, 14%) and *general outpatient clinic* (1/7, 14%). Pictures and texts were the principal media of practicing telemedicine and were used by all 7 hospitals. But, telephone consultation was feasible in 2 (29%) hospitals, and video consultation was applied in 1 (14%) hospital. Service price was uniform in 3 (43%) hospitals, whereas it was positively associated with doctors’ professional titles in the other 3 (43%) hospitals. Only 2 (29%) TCM internet hospitals currently support payment through China’s medical insurance program.

**Table 4.** Telemedicine provided by traditional Chinese medicine (TCM) internet hospitals.

Name of the internet hospital	Name of telemedicine	Content of telemedicine	Service media of telemedicine	Service prices of telemedicine	Service guidelines of telemedicine	Payment methods of telemedicine
Internet Hospital–The First Affiliated Hospital of Guangzhou University of Chinese Medicine	Web-based clinic	Prescription refills for patients with subsequent visits, and web-based consultations on noncritical diseases	Picture and text	RMB ¥10^a^ per web-based visit	—^b^	Patient self-payment
Internet Hospital–Guangdong Second TCM Hospital	Web-based diagnosis and treatment	Interpretation of medical reports, prescription refills for patients with chronic disease and subsequent visits, web-based consultations on noncritical diseases	Picture and text	RMB ¥10 per web-based visit	Doctors respond to consultation questions of patients within 48 hours.	Patient self-payment
Internet Hospital–Guangdong Provincial Hospital of Chinese Medicine	General outpatient clinic	Follow-up of patients who primary visits to the physical hospital	Picture and text Telephone	Chief doctor: RMB ¥30 per web-based visit Associated chief doctor: RMB ¥20 per web-based visit Attending doctor RMB ¥10 per web-based visit	Consultations in picture and text allow unlimited communications between the doctor and the patient within 24 hours. Telephone consultation is limited to 15 minutes.	Guangzhou City General Medical Insurance Mente Medical Insurance^c^ Group 1
Internet Hospital–The Third Affiliated Hospital of Guangzhou University of Chinese Medicine	Convenient medical service	Interpretation of medical reports, prescription refills for patients with chronic disease and subsequent visits, and web-based consultations on noncritical diseases	Picture and text	RMB ¥10 per web-based visit	Doctors respond to questions of patients within 48 hours.	Patient self-payment
Internet Hospital–Integrated Hospital of TCM, Southern Medical University	Expert clinic	Interpretation of medical reports, prescription refills for patients with subsequent visits, and web-based consultations on noncritical diseases	Picture and text	Chief doctor: RMB ¥60 per web-based visit Associated chief doctor: RMB ¥40 per web-based visit Attending doctor: RMB ¥20 per web-based visit	Patients can send 10 messages as the maximum within 48 hours to communicate with the doctor.	Patient self-payment
Internet Hospital- Clifford Hospital	Medical insurance covering follow-ups and Prescription Refills	Prescription refills and web-based consultations on chronic diseases and noncritical conditions	Picture and text	Free for picture and text consultation Web-based consultations from chief doctor: RMB ¥40 per web-based visit Associated chief doctor: RMB ¥30 per web-based visit Attending doctor: RMB ¥20 per web-based visit	—	Patient self-payment Guangzhou City General Medical Insurance for designated hospitals Mente Medical Insurance for 25 diseases in Group 1 Mente Medical Insurance for 28 diseases in Group 2 in designated hospitals Mente Medical Insurance for psychiatric diseases
Internet Hospital–The Affiliated TCM Hospital of Guangzhou Medical University	Web-based clinic	Web-based consultations and chronic disease management	Picture and text Telephone and video	—	The hospital arranges shifts for doctors.	Patient self-payment

a A currency exchange rate of RMB ¥1=US $0.1409 is applicable.

b —: missing information.

c Mente Medical Insurance: a medical insurance program for chronic diseases (coronary heart disease, hypertension, diabetes, epilepsy, and cancer.) requiring long-term and expensive outpatient treatment. The chronic diseases covered by Mente Medical Insurance are classified into 2 groups depending on disease severity. Group 1 is common chronic disease such as, hyperthyroidism, and tuberculosis; and Group 2 was composed of severer diseases, for example leukaemia and renal failure.

Telepharmacy aimed to guarantee drug supply and instructed patients on rational medication use. It consisted of “prescription refills,” “self-service drug purchase,” and “consultation on drug use.” Refilled prescriptions were available as hospital preparations and ointments. These medications can be delivered to patients either through patients collecting drugs from physical hospitals or by a third-party mailer.

Telenursing comprised services of *nursing clinic* and *home-based nursing.* Professional instructions on dealing with common traumas and operations of medical devices can be accessed in *nursing clinic*. *Home-based nursing* supported appointments from patients after surgery and seeking out-reaching rehabilitation services. Web-based health consultation service was health promotion principally focusing on miscellaneous health-related questions. Diagnoses and medical orders were out of the functions of this service item.

Internet hospitals incorporated convenient services to improve patients’ experience during medical services. Differences were in the functions of this service item. Convenient service of the 2 public nonprofit hospitals was mainly about making appointments for offline outpatient visits and medical tests and uploading test reports. Aside from these functions, the other private and for-profit hospitals have an array of different functions, including reporting inpatient medical records, processing of medical insurance, and complaints and suggestions.

#### TCM Specialty Service

TCM specialty services were administered by linking online and offline services. *Tianzhi* (crude herb moxibustion) was one service item of the First Affiliated Hospital of Guangzhou University of Chinese Medicine. Web-based service of *Tianzhi* provided advice on its target population, warnings, prices, and timetables. Processing appointments and payments are also components of the web-based service. The practice of *Tianzhi* will be carried out in the outpatient clinic offline after confirmation of web-based appointments and payments. *Patient-centered* prevention, treatment, and health management were offered by Internet Hospital–The Third Affiliated Hospital of Guangzhou University of Chinese Medicine. This individualized health service is mainly led by renowned TCM practitioners who can identify markers of the onset of disease in a timely manner and develop TCM prevention and treatment programs. The stroke prevention and rehabilitation service offered by Internet Hospital–Integrated Hospital of TCM, Southern Medical University, contained web-based consultation and appointments for acupunctures and massages.

### Comparative Patterns Across Hospital Types and Affiliations

Notable differences were observed across hospital types and ownership. Public nonprofit hospitals primarily focused on providing functional services such as telemedicine and telepharmacy, while their convenient services were limited to basic appointment bookings and report uploads. In contrast, the private for-profit hospital offered a broader range of convenient services, including inpatient record access and medical insurance processing, likely reflecting its market-oriented operational model. Furthermore, comprehensive TCM hospitals tended to deliver a wider variety of web-based services, whereas the specialized TCM hospital emphasized individualized rehabilitation-oriented services. Despite these differences, all hospitals shared common value offerings related to improving patient compliance and promoting TCM awareness.

## Discussion

### Principal Findings

In this study, we conducted an in-depth qualitative analysis of the early-stage digital adaptation in TCM internet hospital health services regarding target patient, value offering, and service provision. All the sampled TCM internet hospitals were established based on Grade A tertiary brick-and-mortar hospitals supported by sufficient health resources. Service content was categorized as functional service (telemedicine, telepharmacy, telenursing, web-based health consultations, and convenient service) and TCM specialty service. All these categories were at the early stage of development. Due to limitations in TCM therapeutic items, TCM specialty services are not prominent in health services. TCM internet hospital has not absorbed the benefits of digitalization from web-based platforms. Current health services have displayed 2 types of value offerings: TCM promotion and optimization of patients’ compliance.

Although this study initially used an *innovation* framing, the empirical findings largely describe early-stage digital adaptation characterized by follow-up consultations and prescription refills. Innovation in this context lies primarily in the reconfiguration of service processes, such as integrating online-offline pathways, linking pharmacy and nursing services, and embedding convenience functions, rather than the introduction of novel digital therapeutics. We therefore present the results as evidence of incremental service evolution with emerging innovative potential, rather than fully realized innovations.

### Practicality of TCM Internet Hospitals

All 7 TCM internet hospitals were established based on physical tertiary hospitals in Grade A, in spite of being for profit or nonprofit. At present, Grade A tertiary hospitals in China have noteworthy advantages in terms of medical equipment, doctors, pharmacists, nurses, and patient flow when compared to hospitals in other grades [25,26]. Therefore, the development of internet hospitals is characterized by relying on sufficient health resources and a large population base.

The overall health services of TCM internet hospitals are at the exploration stage. Telemedicine lacks the initiation of patient-centered interventions and digital therapeutics. The main contents of telepharmacy and telenursing were primary service items, including prescription refills, instructions on managing common traumas, and postsurgery rehabilitation. Convenient service is designed to optimize patients’ experience of web-based medical visits. However, this service was provided by no more than half of the surveyed hospitals. Overall, the limited functional service indicates the significant gap between the current status and the ultimate goal of enhancing the accessibility of health resources and improving patients’ medical experience [27]. Besides, the advantages of digital health are not evident in current web-based health services. Web-based platforms provide the feasibility of medical data storage and management in large quantities and high efficiency, and data sufficiency is the prior condition of patient-centered treatment.

As a part of functional services, telemedicine was mainly for follow-up monitoring on patients’ conditions, interpretation of medical reports, and making subsequent prescriptions. However, the performance of telemedicine may be impacted by web-based modes. Individualized care is emphasized by TCM health services, in which diagnoses were usually performed depending on 4 approaches: *Wang* (observation), *Wén* (auscultation and olfaction), *Wèn* (inquiry), and *Qie* (pulse diagnosis) [28]. However, the general media of telemedicine were pictures and texts. Thus, the *Wén* (auscultation and olfaction) and *Qie* (pulse diagnoses) were restricted. *Wang* (observation) can be influenced by the discretions of picture quality.

Obviously, polarization was in the characteristics of telemedicine, including service prices and payment methods in all the 7 TCM internet hospitals. It is challenging to manage telemedicine service prices, with various service items [29]. From the 7 TCM internet hospitals, 3 (43%) hospitals charged patients RMB ¥10 uniformly, despite different service items and professionals of doctors on shift. In contrast, the other 3 (43%) TCM internet hospitals determined the service prices based on doctors’ titles (chief, associated chief, and attending doctors). The uniform service fees of RMB ¥10 cannot afford frequent manpower- and time-consuming web-based service and technical support for platforms, which can impact the doctors’ proactivity in participation [30]. A majority (5/7, 71%) of the internet hospitals did not support National Medical Insurance, a potential barrier for patients to web-based services. Incorporating web-based diagnosis and treatment items with high prevalence into the payment scope of the National Medical Insurance Catalog helps improve general service quality, thereby satisfying patients’ growing needs in the health care [31].

In addition, hospital ownership and type influenced the scope of web-based service delivery. Public nonprofit hospitals provided more standardized but narrower functional services, whereas the private for-profit hospital adopted a more market-driven approach by expanding convenient services. Comprehensive hospitals integrated broader service items compared to specialized hospitals, which focused more on niche rehabilitation services. These nuanced patterns suggest that organizational structure and operational models may shape service early-stage digital adaptation trajectories in TCM internet hospitals.

### TCM Specialty Service

Offline TCM services are various and involve pharmacological interventions, including Chinese patient medicine, Chinese herbal piece, Chinese herbal medicine formula granule, and Chinese herbs; and nondrug interventions, including dietary therapy, acupuncture, moxibustion, massage, cupping, scraping, and therapeutic mind or body practices [32,33]. TCM has been approved as effective in treating chronic diseases, including tumors [34], diabetes [35], and cardiovascular diseases [36]. Clinical evidence of TCM has also been found in treating skin diseases and infertility [37,38]. China is the sole country that provides Western medicine and TCM services at hospitals and medical institutions at different levels [39]. However, judging from the use of health resource consumption in TCM hospitals, the role of TCM is still unclear due to the increasing influence of Western medicine. The TCM service system requires improvement [40].

Internet hospitals can contribute to the refinement of the TCM service system. With the rapid development and application of information technologies such as the internet, big data, artificial intelligence (AI), and the web of things in the health system, digitalization is accelerating in the TCM [41]. In October 2019, the State Council issued the “Opinions on Promoting the Inheritance and Innovative Development of Traditional Chinese Medicine,” which encourages the establishment of TCM internet hospitals by relying on physical hospitals, development of intelligent auxiliary systems for disease diagnosis and treatment, implementation of telemedicine, and integration of web-based and offline TCM services [42]. Currently, clinical decision support systems have been upgraded with AI techniques. Examples of the systems are pulse diagnosis and tongue diagnosis systems. They are gradually applied to web-based consultations to assist TCM practitioners in understanding patients’ health conditions [43,44].

In the meantime, TCM specialty service is amenable to being the characteristic service of TCM internet hospitals. However, its performance is not prominent. Items of TCM specialty service were *Tianzhi* (crude herb moxibustion) [45], *Zhiweibing* (preventive treatment of disease) [46], and stroke rehabilitation. Roles of the items were mainly health consultations and preservations, with the absence of therapeutics. TCM therapeutics, such as phlegm scraping, massage, and acupuncture [8], have been widely applied offline but are augmented by *internet +*.

Despite the unique modalities of TCM, most internet hospital functions remain generic, centered on teleconsultations and prescription refills. Distinctive services (eg, acupuncture, tuina, moxibustion, and rehabilitation) were rarely offered web-based. This mismatch reflects three constraints: (1) technical limitations of remote media, particularly in replicating pulse-taking and olfactory diagnosis; (2) regulatory and reimbursement restrictions, which limit insurance coverage for specialty treatments; and (3) the absence of standardized digital workflows and quality control mechanisms. Addressing these barriers requires a staged roadmap: in the short term, establishing a hybrid “web-based assessment–offline treatment” pathways; in the medium term, expanding reimbursement and pricing policies to include distinctive TCM modalities; and in the long term, integrating AI-assisted tongue or pulse diagnostics and digital biomarkers into web-based TCM platforms.

### Potential Orientations to Future Innovations

According to the *Internet Hospital Management Measures (Trial)* policy [47], telemedicine provided by internet hospitals should be restricted to patients with follow-up visits for common and chronic diseases. In addition to this basic function, TCM internet hospitals are carrying out items of TCM health consultations and preservations. These items are critical for fulfilling the general population’s health needs, which is consistent with the policy of *Outline of the 14th Five-Year Plan for National Economic and Social Development of the People’s Republic of China and the vision 2035* [48]. Meanwhile, TCM has been well applied for rehabilitation in the whole health system, especially in primary care [49]. Therefore, the additive service items offered by TCM internet hospitals are an advance in both web-based and TCM services. In addition, TCM internet hospitals are indicated as optimal platforms for people in need of disease prevention and health protection. In the overall hierarchical medical system, TCM internet hospitals can provide first-line health management for people before they get ill and access primary services with definite symptoms. On this note, TCM internet hospitals contribute to popularizing and developing TCM.

Compliance is a noteworthy factor affecting the quality and efficiency of medical services. Inadequate compliance is significantly associated with increased mortality, morbidity, and medical costs [50-52]. Education, reminders, and financial incentives to patients can improve their compliance [53]. Common factors impeding medical services are high costs, limited access, and long duration [22], all present in offline service scenarios. TCM internet hospitals can remove these obstacles, thereby improving medical compliance. The findings of interviews have reflected this proposition.

In addition, the collection and adoption of digital biomarkers are potentially beneficial from the platform of TCM internet hospitals. Biomarkers are objective, quantifiable medical signs that relate to medical signs, symptoms, surrogate end points, or clinical end points [54]. RockHealth defined digital biomarkers as “consumer-generated physiological and behavioral measures collected through connected digital tools that can be used to explain, influence, and predict health-related outcomes” [55]. In the era of digital health, digital biomarkers enable new value chains on health data for health intervention [56]. Developing and adopting safe and effective digital biomarkers to improve patient outcomes in clinical research and routine patient care has been a new trend [57]. As an easily scalable, noninvasive complement, a smartphone can provide readily attainable digital biomarkers of health intervention, such as diabetes and Alzheimer disease [58,59]. As TCM specialty services are incorporated into TCM internet hospitals for health consultations and preservations, they can potentially become a novel path in collecting digital biomarkers, such as user-generated measurements. These biomarkers are sources of evidence and valid decision-making. In this study, TCM internet hospitals primarily targeted chronic disease follow-ups and health preservation consultations. These service patterns provide practical application scenarios where AI-driven decision support and digital biomarkers could enhance long-term patient monitoring and personalized TCM interventions, aligning with the observed patient needs.

### Comparison With Prior Research and Theoretical Implications

Our findings corroborate prior studies indicating that internet hospitals improve accessibility and continuity of care for patients with chronic and stable conditions [2,4]. However, unlike previous research primarily focused on Western medicine internet hospitals, we identified an additional value dimension in promoting TCM literacy and health preservation practices, aligning with studies that emphasize TCM’s preventive care philosophy [14,15]. Furthermore, while earlier work suggested that digital platforms primarily address service inefficiencies [11], our study demonstrates their potential to enhance patient compliance and extend TCM specialty services such as crude herb moxibustion and stroke rehabilitation.

From a theoretical perspective, the findings enrich the *value proposition* element of the business model framework by Johnson et al [22]. Specifically, TCM internet hospitals create a dual-layered value: they not only address patients’ immediate medical needs but also extend cultural and educational functions unique to TCM. This differs from the more transactional value propositions of typical internet hospitals, suggesting that TCM internet hospitals may operate under a hybrid value model combining clinical and cultural components.

In contrast to prior work highlighting limited integration between web-based and offline services [16], our study reveals emerging hybrid models that link web-based consultations with offline therapeutic interventions. This finding extends the understanding of digital health adoption by illustrating how TCM internet hospitals bridge digital health technologies with traditional therapeutic modalities.

### Implications for Policy and Practice

Overall, the development of web-based TCM health services is in the early stage with functional service occupying a large proportion. There was a remarkable gap between the current health service and an advanced model with prominent TCM therapeutics, generalized convenient service, and full integration of digital technology. The value proposition of TCM internet hospitals is yet to be excavated, aside from current assistance with the promotion of TCM and patients’ compliance. Challenges in developing TCM health services are various, including heterogeneous qualifications of practitioners, difficulties in obtaining patients’ medical records, lagging establishment of guidelines and legal terms for web-based practice, and insufficient programs for privacy protection [60]. Several suggestions can be made to overcome these challenges. First, more TCM therapeutics are expected, which include acupuncture, cupping, and scraping. It is necessary to formulate and implement nationwide guidelines for web-based medical practice along with the expansion of service items. Second, it is important to enhance convenient service to refine the patients’ experience. Third, schemes of rationing internet service prices and health insurance coverage of service items are critical for upgrading health care for patients and the proactivity of doctors’ participation in web-based services. Finally, it is suggested to make use of the digital benefits of web-based platforms and encourage TCM internet hospitals to develop and adopt safe and effective digital biomarkers to improve patient outcomes. To ensure privacy and autonomy, data use agreements for digital biomarkers should contain clear statements on data use conditions, especially for near-continuous data, such as movement, voice, and other sensitive biometric states.

However, further innovation is constrained by uneven IT infrastructure, regulatory uncertainties, and limited insurance coverage for web-based TCM services. To address these barriers, policymakers should promote standardized IT infrastructure and data-sharing protocols, improve regulatory clarity for web-based TCM consultations, and expand insurance coverage for commonly used TCM digital services. In parallel, health care institutions should develop clinical guidelines for digital TCM care to enhance provider confidence and patient trust. These coordinated efforts could create a more supportive ecosystem for innovation and sustainable scaling of TCM internet hospitals.

In practice, short-term priorities include standardizing telemedicine workflows and improving IT interoperability at the hospital level. Medium- to long-term priorities require coordinated policy action, such as expanding insurance coverage for web-based TCM services and clarifying regulatory frameworks for digital TCM practice. These recommendations are particularly urgent in the context of China’s ongoing digital health reforms, including the nationwide expansion of internet hospital regulation and health insurance integration pilots. Aligning the TCM internet hospitals with these reforms—through standardized IT systems, clearer liability frameworks, and targeted reimbursement policies—would ensure they are not marginalized but become an integral part of the evolving digital health ecosystem.

### Strengths and Limitations

To the best of our knowledge, this study is the first effort to investigate early-stage digital adaptation in health services of TCM internet hospitals. We used a combination of several qualitative methodologies, including case studies, stakeholder interviews, and document analysis, to fulfill the aim. However, there are several research limitations that cannot be ignored. First, a key limitation is that all participating hospitals were located in Guangzhou. While Guangzhou represents an early and advanced hub for TCM digital services, findings may not fully reflect practices in less developed provinces or rural settings. Accordingly, the results should be interpreted as contextual and transferable insights rather than nationally representative estimates. Future research should extend to multi-provincial and urban–rural comparative samples. Second, this study investigated early-stage digital adaptations in TCM health services by interviewing service practitioners. Another limitation is the absence of patient perspectives, which restricts the ability to fully assess service acceptability and satisfaction. To address this, future studies will incorporate patient satisfaction surveys, semistructured interviews, and usage data (such as revisit rates, prescription completion, and delivery timeliness), enabling a more balanced evaluation of TCM internet hospital services. Thirdly, this study lacked analysis on the service quality and cost-effectiveness of TCM internet hospitals. Future research should conduct extensive quantitative analysis to assess the service quality and cost-effectiveness of TCM internet hospitals and compare them to traditional TCM hospitals. Fourth, this study relied on self-reported interviews with clinicians, which may be subject to recall and social desirability bias. The findings primarily reflect providers’ perspectives and may not fully capture objective service quality. Future research could triangulate interview data with patient feedback, service usage metrics, and quantitative quality indicators to enhance validity. Finally, future research should consider investigating the integration of AI and big data analytics in TCM internet hospital services.

Overall, this study advances the current understanding of digital health services by situating TCM internet hospitals within both the local cultural context and broader digital health transformation. By explicitly linking patient compliance, TCM promotion, and hybrid service delivery models, our findings extend prior work and provide empirical evidence to refine theoretical models of digital health innovation, particularly in resource-integrated traditional medicine settings. While the findings reflect the unique cultural and policy context of China’s health care system, certain insights—such as the role of internet hospitals in chronic disease follow-up and preventive health consultations—may be transferable to other countries exploring the digitalization of traditional or complementary medicine. However, the scalability of TCM internet hospitals in other contexts would depend on local regulatory frameworks, health insurance structures, and the cultural acceptance of traditional medicine. Future comparative studies are needed to assess the adaptability of this model in different health care systems.

### Conclusions

This study positions TCM internet hospitals as a potentially disruptive force in the early stage of technology adoption and diffusion. While they currently focus on serving returning patients with chronic or stable conditions and promoting TCM health preservation, the innovative potential of TCM-specific services remains underexplored. To truly become a positive disruption that strengthens TCM specialty services and enhances patient accessibility, TCM internet hospitals must transition from the early adoption phase to the early majority stage.

Our findings enrich the understanding of how traditional medicine integrates with digital health, highlighting the hybrid value proposition of TCM internet hospitals that combines clinical care with cultural and educational functions. They also reveal systemic barriers—uneven IT infrastructure, regulatory uncertainties, and limited insurance coverage—that constrain innovation diffusion, emphasizing the need for coordinated policy and institutional reforms. For stakeholders, these insights provide a roadmap to better harness digital health innovation in TCM. Future research should triangulate provider and patient perspectives with platform data to validate service quality and explore the integration of AI-driven decision support and digital biomarkers to personalize TCM care and accelerate sustainable scaling.

## Supplementary material

10.2196/77686 Multimedia Appendix 1 Informed consent.

10.2196/77686 Multimedia Appendix 2 Interview schedule.

## Abbreviations

AI: artificial intelligence
TCM: traditional Chinese medicine

## References

[1] Liang F, Yang X, Peng W (2024). Applications of digital health approaches for cardiometabolic diseases prevention and management in the Western Pacific region. Lancet Reg Health West Pac.

[2] Tu J, Wang C, Wu S (2015). The internet hospital: an emerging innovation in China. Lancet Glob Health.

[3] Gu Y, Wei D, Cao X, Zhang C, Feng X (2017). Analysis of the reason of the internet hospital occurred in China. Chinese Health Serv Manag.

[4] Lai Y, Chen S, Li M, Ung COL, Hu H (2021). Policy interventions, development trends, and service innovations of internet hospitals in China: documentary analysis and qualitative interview study. J Med Internet Res.

[5] Wang L, Suo S, Li J (2017). An investigation Into traditional Chinese medicine hospitals in China: development trend and medical service innovation. Int J Health Policy Manage.

[6] Tang JL, Liu BY, Ma KW (2008). Traditional Chinese medicine. Lancet.

[7] Tian J, Gao S (2022). Analysis on barriers and strategies of the development of “internet+” traditional Chinese medicine treatment. Health Econ Res.

[8] Hu T, Zhou B, Ling Z, Chen Z, Chen C, Zhu J (2022). Study on the construction status and development trend of internet traditional Chinese medicine hospitals. J Med Inform.

[9] Wang C, Li R, Yan K, Ma S (2023). High quality development of traditional Chinese medicine hospitals based on the comparison of medical resources and medical service. Chin Health Qual Manag.

[10] Ma H, Xiong J (2023). Study on spatial distribution and influencing factors of service efficiency of TCM hospitals. Soft Sci Health.

[11] Wang X, Liu K, Zhang H, Li X, Ma Z (2021). Research and practice on internet service mode of TCM diagnosis and treatment. Chin J Health Inform Manag.

[12] Zhang Y, Tian Y, Feng JJ, Liu JW, Zhang YL, Lin LK (2024). Development situation and expert suggestion on “Internet + Traditional Chinese Medicine” in China. New Med.

[13] Wang X, Chen J, Feng M (2023). Demand and influencing factors of “Internet + Traditional Chinese Medicine” home nursing service for older adult patients with chronic diseases: a mixed research perspective. Front Public Health.

[14] Zhang L, Zhou PY, Tang H (2023). Practice and exploration of independent construction of internet hospitals of traditional Chinese medicine hospitals. Smart Healthc.

[15] Qi MJ, Chen KY, Liu ZX (2024). Online medical services by Beijing hospital of traditional Chinese medicine. Modern Hosp.

[16] Han YY, Yuan M, Jiang H, Yu J, Tang WJ (2024). Service and practice of internet hospital established in a large public traditional Chinese medicine hospital. Chin J Med Manag Sci.

[17] Qi MJ, Zhang QS, Han YL, Chen KY, Dong JC (2023). A survey on use of internet plus health care among medical staff in a grassroots traditional Chinese medicine hospital. Chin J Med Manag Sci.

[18] Ding YW, Zhai X, Wang QX, Tong JD, Kou JM (2022). Status quo of the information services of mobile medical apps for traditional Chinese medicine. Chin J Med Libr Inf Sci.

[19] Ge Y, Yao D, Ung COL (2023). Digital medical information services delivered by pharmaceutical companies via WeChat: qualitative analytical study. J Med Internet Res.

[20] Ding Y, Zhai X, Wang Q, Kou I, Tong J (2022). Research on the construction of evaluation index system of information service quality of Internet traditional Chinese medicine hospital. Chin Hosp.

[21] Shu Y, Ning L, Chen L, Huang F, Wu W (2021). Practice and thought on the establishment of internet hospitals by public Chinese medicine hospitals. Chin Hosp.

[22] Johnson MW, Christensen CM, Kagermann H (2008). Reinventing your business model. Harvard Bus Rev.

[23] Lai Y, Ho Q, Wang Y (2024). Challenges and strategies of developing internet hospital: combining qualitative interview and documentary research. Digit Health.

[24] (2022). Grade a tertiary hospital accreditatin standards. National Health Commission of the Peopole’s Republic of China.

[25] Lv Q, Jiang Y, Qi J (2019). Using mobile apps for health management: a new health care mode in China. JMIR Mhealth Uhealth.

[26] Zhang X, Zhang X, Yang S, Wang Y (2019). Factors influencing residents’ decision to sign with family doctors under the new health care reform in China. Int J Health Plann Manage.

[27] Zhang M, Wang Y, Qian Z, Wang D (2019). Research on development modes of internet hospitals in China. Health Econ Res.

[28] Chen Q, Zhang G, Zhang H, Wang C, Zhang K (2009). Progress of application of modern information technology in four diagnostic methods. Jiangxi J Tradit Chin Med.

[29] Liu J (2020). Difficulities and countermeasures for our internet medical service price management. Prices Mon.

[30] Zheng D, Nie L, Wang L, Chen Y (2021). Medical insurance management under the background of ’Internet+Medical’. Chin Hosp.

[31] Pan Y, Yang Y, Xia Y (2023). An analysis of the effect of “internet+” medical services incorporated into medical insurance payment in tertiary general hospitals in Shanghai. Modern Hosp.

[32] Xiao LJ, Tao R (2017). Traditional Chinese medicine (TCM) therapy. Adv Exp Med Biol.

[33] Meng F, Ye M, Si J (2022). Status of traditional Chinese medicine healthcare services in nursing homes across China. Geriatr Nurs.

[34] Wang K, Chen Q, Shao Y (2021). Anticancer activities of TCM and their active components against tumor metastasis. Biomed Pharmacother.

[35] Martel J, Ojcius DM, Chang CJ (2017). Anti-obesogenic and antidiabetic effects of plants and mushrooms. Nat Rev Endocrinol.

[36] Feng X, Sureda A, Jafari S (2019). Berberine in cardiovascular and metabolic diseases: from mechanisms to therapeutics. Theranostics.

[37] Yan F, Li F, Liu J (2020). The formulae and biologically active ingredients of Chinese herbal medicines for the treatment of atopic dermatitis. Biomed Pharmacother.

[38] O’Reilly E, Sevigny M, Sabarre KA, Phillips KP (2014). Perspectives of complementary and alternative medicine (CAM) practitioners in the support and treatment of infertility. BMC Complement Altern Med.

[39] Xu J, Yang Y (2009). Traditional Chinese medicine in the Chinese health care system. Health Policy.

[40] Shi X, Zhu D, Nicholas S, Hong B, Man X, He P (2020). Is traditional Chinese medicine “mainstream” in China? Trends in traditional Chinese medicine health resources and their utilization in traditional Chinese medicine hospitals from 2004 to 2016. Evid Based Complement Alternat Med.

[41] Wang X, Xie Y, Yang X, Gu D (2023). Internet-based healthcare knowledge service for improvement of Chinese medicine healthcare service quality. Healthcare (Basel).

[42] (2019). Opinions on facilitating the inheritance, innovation and development of traditional Chinese medicine. The State Council of the People’s Republic of China.

[43] Tang ACY, Chung JWY, Wong TKS (2012). Digitalizing traditional Chinese medicine pulse diagnosis with artificial neural network. Telemed J E Health.

[44] Wang X, Liu J, Wu C (2020). Artificial intelligence in tongue diagnosis: Using deep convolutional neural network for recognizing unhealthy tongue with tooth-mark. Comput Struct Biotechnol J.

[45] Pan H (2009). The development and clinical application of “Tianzhi" treatment. Gansu Technol.

[46] Jing Q (2018). Preventive treatment in traditional Chinese medicine. Med Inform.

[47] (2018). Notice on printing and distributing three documents including the internet medical consultation administrative measures (trial). National Health Commission of the People’s Republic of China, National Administration of Traditional Chinese Medicine of the People’s Republic of China.

[48] (2021). Outline of the fourteenth five-year plan for national economic and social development of the people’s republic of china and the vision 2035. General Office of the State Council of the People’s Republic of China.

[49] Fang L, Zhu RR, Sang Z (2023). World Health Organization survey on the level of integration of traditional Chinese medicine in Chinese health system rehabilitation services. Integr Med Res.

[50] Simpson SH, Eurich DT, Majumdar SR (2006). A meta-analysis of the association between adherence to drug therapy and mortality. BMJ.

[51] Ho PM, Magid DJ, Shetterly SM (2008). Medication nonadherence is associated with a broad range of adverse outcomes in patients with coronary artery disease. Am Heart J.

[52] Ho PM, Rumsfeld JS, Masoudi FA (2006). Effect of medication nonadherence on hospitalization and mortality among patients with diabetes mellitus. Arch Intern Med.

[53] Anderson LJ, Nuckols TK, Coles C (2020). A systematic overview of systematic reviews evaluating medication adherence interventions. Am J Health Syst Pharm.

[54] Strimbu K, Tavel JA (2010). What are biomarkers?. Curr Opin HIV AIDS.

[55] Wang T, Azad T, Rajan R (2016). The emerging influence of digital biomarkers on healthcare. Rock Health.

[56] Meister S, Deiters W, Becker S (2016). Digital health and digital biomarkers—enabling value chains on health data. Curr Dir Biomed Eng.

[57] Coravos A, Khozin S, Mandl KD (2019). Developing and adopting safe and effective digital biomarkers to improve patient outcomes. NPJ Digit Med.

[58] Avram R, Olgin JE, Kuhar P (2020). A digital biomarker of diabetes from smartphone-based vascular signals. Nat Med.

[59] Kourtis LC, Regele OB, Wright JM, Jones GB (2019). Digital biomarkers for Alzheimer’s disease: the mobile/wearable devices opportunity. NPJ Digit Med.

[60] Ye G, Chen J, Zhang S, Yuan H (2023). Research on the current situation, problems and countermeasures of internet medical treatment of traditional Chinese medicine. Chin Hosp.

