# Supplementary material for: Health Service Early-Stage Digital Adaptation of Traditional Chinese Medicine Internet Hospitals: Qualitative Exploratory Study
Source: JMIR Form Res. 2025 Nov 5;9:e77686. doi: 10.2196/77686 (PMC12588593; doi:10.2196/77686)
Supplement: Multimedia Appendix 1 [file formative-v9-e77686-s001.docx]

**Informed Consent**

**Project Title:** Health service innovation of traditional Chinese medicine (TCM) internet hospitals

**Principal Investigator:** Dr. Yunfeng Lai (Guangzhou University of Chinese Medicine), Dr. Hao Hu (ICMS, University of Macau)

**Study Sponsor:** Guangzhou University of Chinese Medicine, University of Macau

**Study Purpose**

This study aimed to investigate the health service innovation of TCM internet hospitals in China. It is expected that the findings from this study can contribute to further innovation of TCM internet hospitals in China and provide references for the international development of internet hospitals for personalized digital health and patient-centric services.

**Study Procedures**

During this study, you will be asked to answer some questions primarily focused on the service innovation of TCM internet hospital. If there are any questions you feel you cannot answer or that you do not feel comfortable answering, there is no obligation for you to answer, and you may withdraw from the study at any time if you wish. The interview will last about 30 to 60 minutes.

**Possible Discomforts or Risk**

There are no known harms or discomforts associated with this study beyond those encountered in normal daily life.

**Benefits**

While you might not directly benefit from participation in this study, your help could contribute to deepen social understanding of the innovation of internet hospital.

**Data Collection, Storage and Usage**

We would like to request your permission to tape-record the interview. However, no identifiable information will be collected. The final data will be stored and accessed by the researchers themselves. All the data will be only used for academic reasons.

**Withdraw**

Participation in this research study is completely voluntary. You are free to withdraw from this study at any time.

**Questions about the Research**

If you have questions regarding this study, you may contact Yunfeng Lai at [laiyunfeng@gzucm.edu.cn](mailto:laiyunfeng@gzucm.edu.cn) and Hao Hu at [haohu@umac.mo](mailto:haohu@umac.mo)

***_____________________________________________________________________________________***

**Subject’s Understanding**

- I understand the research purpose and procedures.
- I understand that my participation is voluntary.
- I understand that all data collected will be limited to this use or other research-related usage as authorized by Guangzhou University of Chinese Medicine and University of Macau.
- I understand that I will not be identified by name in the final report or other project publications.
- I am aware that all records will be kept confidential in the secure possession of the researcher.
- I acknowledge that the contact information of the researcher has been made available to me along with a duplicate copy of this consent form.
- I understand that I may withdraw from the study at any time.

I have read the above form, and, with the understanding that I can withdraw at any time, and for whatever reason, I consent to participate in this interview. [Yes/No] _______________________

**Tape-recording**

- I agree for my interview to be tape-recorded. [Yes/No] __________________

_____________________ ___________________

Participant’s signature Date

____________________ ___________________

Interviewer’s signature Date

**知情同意书**

**项目名称：**中医互联网医院的健康服务创新研究

**项目负责人**：赖云锋博士（广州中医药大学）、胡豪博士（澳门大学）

**赞助方：**广州中医药大学、澳门大学

**研究目的**

这项研究的目的是探讨中国中医互联网医院的健康服务创新。以期为中国中医互联网医院的进一步创新做出贡献，为国际互联网医院发展数字化健康医疗及以患者为中心健康服务提供参考。

**研究程序**

在这项研究中，您会被要求回答一些问题，关于您对中国中医互联网医院的服务创新的思考与观点。如有任何问题让您觉得不适或无法回答，您无需承担责任，可以在任何时间退出这项研究。整个访谈约持续30-60分钟。

**可能的不适或风险**

本研究没有超出日常生活外的已知相关危害或不适。

**效益**

虽然您可能无法直接从本次研究中获益，您的帮助有助于加深社会对互联网医院政策干预和服务创新的理解。

**数据的收集，存储和使用**

我们想请您同意录音记录采访。但是，没有收集可识别身份的信息。最终的数据将由研究者存储和读取。所有的数据将只用于学术研究。

**退出**

这项研究完全是自愿参与。您可以随时退出这项研究。

**研究的相关问题**

如果您有任何关于这项研究的问题，您可以联系赖云锋（[laiyunfeng@gzucm.edu.cn](mailto:laiyunfeng@gzucm.edu.cn)）和胡豪（[haohu@umac.mo](mailto:haohu@umac.mo)）

_____________________________________________________________________________________

**同意事项**

- 我了解研究目的和程序。
- 我明白我的参与是自愿的。
- 我知道收集的所有数据将只用于广州中医药大学和澳门大学的此研究项目或相关研究。
- 我明白姓名不会在最后的报告或其它项目的出版物识别。
- 我知道，所有记录都由研究人员保密。
- 我承认已经从研究员的联系信息中取得知情同意书的副本。
- 我知道我可以随时退出研究。

我已阅读上述表格，并知道不管原因，我可以随时退出，我同意参加这次采访。[是/否]____________

**访谈录音**

- 我同意访谈录音。[是/否]______________

_____________________ ___________________

参与者的签名 日期

____________________ ___________________

采访者的签名 日期
