# Supplementary material for: Health Service Early-Stage Digital Adaptation of Traditional Chinese Medicine Internet Hospitals: Qualitative Exploratory Study
Source: JMIR Form Res. 2025 Nov 5;9:e77686. doi: 10.2196/77686 (PMC12588593; doi:10.2196/77686)
Supplement: Multimedia Appendix 2 [file formative-v9-e77686-s002.docx]

**Interview Schedule of Research on the Health service innovation of traditional Chinese medicine (TCM) internet hospitals**

1. **Introduction**

In China, TCM hospitals are accelerating the development of internet hospitals to provide health services to patients. However, research on health service innovation in TCM internet hospitals is very limited. What it the service model of TCM internet hospital? What are the service contents and innovations of TCM internet hospitals? There is currently no systematic evidence to provide reference.

Therefore, this study used key stakeholder interviews to investigate the health service innovation of TCM internet hospitals in terms of service content and value proposition. It is hoped to provide empirical reference for personalized digital health services and patient-centered services in TCM internet hospitals, thereby promoting the inheritance and development of TCM.

**Note: This research is purely academic and does not involve any commercial interests or ethical conflicts.**

1. **Methods**
   1. **Research Design**

Qualitative interviews with participant

- 1. **Interviewee**

Medical workers who provide health services in TCM Internet hospitals.

- 1. **Interview Outline**
     1. **Overview of providing health services in TCM internet hospitals**

1. Which types of patients do you mainly provide services in the TCM internet hospital?
2. What health services do you mainly provide in the TCM internet hospital?
3. What do you think are the differences between online health services in TCM internet hospitals and traditional offline health services?
4. How often do you provide health services in the TCM internet hospital?
   - 1. **Elements of TCM internet hospital health services**
5. What health needs do you think TCM internet hospitals can mainly solve for patients?
6. What health services do patients mainly hope to receive when using TCM internet hospitals?
7. What are the main factors you consider when providing health services based on TCM internet hospitals?
   - 1. **Challenges of TCM internet hospital health services**
8. What challenges do you encounter in providing health services in TCM internet hospitals?
   - 1. **Suggestions and expectations for TCM internet hospital health services**
9. What other health services do you think TCM internet hospitals can provide?
10. How do you think the health services of TCM internet hospitals should be optimized and improved?
11. How to further improve TCM specialty services of TCM internet hospitals?
